# Who Benefits from Ecosystem Services? A Case Study for Central Kalimantan, Indonesia

**DOI:** 10.1007/s00267-015-0623-9

**Published:** 2015-10-14

**Authors:** Aritta Suwarno, Lars Hein, Elham Sumarga

**Affiliations:** Environmental System Analysis Group, Wageningen University, PO Box 47, 6700 AA Wageningen, The Netherlands; School of Life Science and Technology, Institut Teknologi Bandung (ITB), Jalan Ganeca 10, Bandung, 40132 Indonesia

**Keywords:** Ecosystem services, Beneficiaries, Benefits, Ecosystem accounting, Ecosystem management

## Abstract

There is increasing experience with the valuation of ecosystem services. However, to date, less attention has been devoted to who is actually benefiting from ecosystem services. This nevertheless is a key issue, in particular, if ecosystem services analysis and valuation is used to support environmental management. This study assesses and analyzes how the monetary benefits of seven ecosystem services are generated in Central Kalimantan Province, Indonesia, are distributed to different types of beneficiaries. We analyze the following ecosystem services: (1) timber production; (2) rattan collection; (3) jelutong resin collection; (4) rubber production (based on permanent agroforestry systems); (5) oil palm production on three management scales (company, plasma farmer, and independent smallholder); (6) paddy production; and (7) carbon sequestration. Our study shows that the benefits generated from these services differ markedly between the stakeholders, which we grouped into private, public, and household entities. The distribution of these benefits is strongly influenced by government policies and in particular benefit sharing mechanisms. Hence, land-use change and policies influencing land-use change can be expected to have different impacts on different stakeholders. Our study also shows that the benefits generated by oil palm conversion, a main driver for land-use change in the province, are almost exclusively accrued by companies and at this point in time are shared unequally with local stakeholders.

## Introduction

Ecosystem services (ES) are increasingly recognized as a concept that can be used to assess the benefits humans derive from ecosystems in support of ecosystem management (Millennium Ecosystem Assessment (MA) 2005). The concept of ES is broadly defined as the contributions of ecosystems to economic and other human activity (TEEB 2010; UN et al. 2014; Haines-young and Potschin 2013). Benefits from ES are not just a function of ecosystem dynamics but also a function of the socio-economic system (i.e., governance system, markets, and informal land use) (Fisher et al. 2008a). Identification of benefits and beneficiaries from ES is paramount to identify enhanced ecosystem management options (Kettunen et al. 2009).

Several studies have described the concept of beneficiaries and stakeholders of ES for spatial range and specific ecological and economic processes (Hein et al. 2006; TEEB 2010; Bagstad et al. 2014). Studies on how ES benefits received by beneficiaries are altered due to land-use change in several countries have also been conducted from a regional (Tomich et al. 2004; Law et al. 2014) to global scale (Lambin et al. 2003; Howe et al. 2014). However, there is still insufficient insight in how different stakeholders benefit from different types of ES and what this means for ecosystem management (Daily et al. 2009)

The objective of our study is to analyze the benefits of seven ES in Central Kalimantan Province, Indonesia, and to examine how these benefits are distributed to different types of beneficiaries. Our study was conducted in three steps: First, we defined the beneficiaries based on the spatial range of ES, related to specific ecological and economic processes (Hein et al. 2006; Bagstad et al. 2014). Second, we calculated the monetary benefits of ES based on ecosystem accounting (UN et al. 2014). Third, we analyzed the benefits received by different types of beneficiaries among others based on existing government regulations in the forestry and agricultural sectors. Further, we analyzed the potential gains and losses of land-use changes through the calculation of total benefits of ES and the estimation of damage costs of CO_2_ emissions (Interagency Working Group on Social Cost of Carbon 2013).

We use the ecosystem accounting framework as the methodological framework for our study. Ecosystem accounting is a new area of environmental economic accounting, which aims to measure ecosystem capital in a way that is consistent with national accounts (Boyd and Banzhaf 2007; UN et al. 2014; Edens and Hein 2013). Ecosystem accounting provides a framework for analyzing ecosystem condition, ecosystem service flow, and ecosystem assets, using a set of physical and monetary indicators. This approach analyzes the monetary value of production and consumption based on exchange values at ‘arm’s length.’ Contrary to welfare-based valuation approach, it does not include consumer surplus.

The innovative aspects of our study are (1) the implementation of an ecosystem accounting approach to determine the monetary benefits of ES received by the different groups of beneficiaries and (2) linking this information to support ecosystem management. Given the importance of ES benefits in supporting ecosystem management, from the results of this study, we aim to provide valuable input to establish ecosystem management in Central Kalimantan Province.

## Methodology

### Study Area

**Fig. 1:**
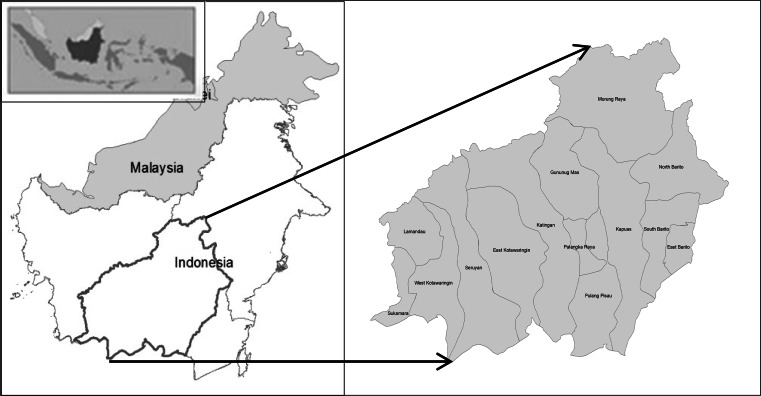
Case study area

This study was conducted in Central Kalimantan Province, Indonesia (Fig. 1). The province covers an area of approximately 15.4 million ha of which 12.7 million ha is designated forest (Ministry of Forestry 2011). The total population in 2010 was 2.15 million, with a population density of 14 people/km^2^. In terms of local GDP, forest and agriculture (particularly oil palm) are the most important sectors. The forests and peatlands of Central Kalimantan are part of the biodiversity hotspot of Borneo’s forest and believed to be among the most species-rich environments in the world (Whitten et al. 2004). They provide vital ecosystem benefits on a local, regional, and global scale including livelihood products (e.g., timber and non-timber products) (Meijaard et al. 2013); cultural services (e.g., nature recreation) (Hernández-Morcillo et al. 2013; Plieninger et al. 2013); and regulating services (e.g., storage of vast amounts of carbon stock) (Paoli et al. 2010; Leh et al. 2013). However, rapid deforestation to further agricultural and silvicultural development, particularly oil palm, in Central Kalimantan has been a salient issue over the last decade. From 2000 to 2008, the province lost approximately 0.9 million ha of forest (Koh et al. 2011; Broich et al. 2011b). Some studies indicated the expansion of oil palm plantation as the main driving factor of deforestation in this province (Boer et al. 2012; Koh et al. 2011). The oil palm expansion in Central Kalimantan Province has been one of the fastest in Indonesia in the period 2000–2010 (Broich et al. 2011a; Koh et al. 2011; Gunarso et al. 2013).

### Identification of Beneficiaries and Stakeholders

ES stakeholders can be defined as any group of individuals who can affect or are affected by the ecosystem’s service (Hein et al. 2006). ES beneficiaries benefit from ecosystem goods or services either through active or passive consumption, or through simple appreciation resulting from the awareness of these services (Harrington et al. 2010; Nahlik et al. 2012). The distinction between stakeholder and beneficiary is related to the ability to influence ES provision and the dependency on the ES. Each beneficiary should be considered a stakeholder (Hein et al. 2006; Rastogi et al. 2010), but not all stakeholders are necessarily beneficiaries.

The ES benefits vary depending on the type of their individual characteristics, spatial scale, and distance between production area and the location of beneficiaries (Fisher et al. 2008b; Bagstad et al. 2014). In this study, we grouped beneficiaries based on spatial extent and bio-economic process to be consistent with the beneficiaries’ concept in the System of National Accounts (SNA). The beneficiaries of ES are then grouped into (1) private (large companies, small medium enterprises (SMEs), smallholder with hired labor); (2) public (governmental agencies at various levels); and (3) household entities as presented in Table 1.

**Table 1:** Ecosystem services and their key beneficiaries analyzed in this study (adopted from CICES and Haines-young and Potschin 2013)

ES category	ES sub-category	ES benefit domain	Beneficiaries		
			Private (large companies, SMEs, smallholders with hired labor)	Public (government agencies at various levels; global communities)	Household
Provisioning	Traditional agriculture	Upland paddy production			Paddy farmer
		Rubber production under permanent agroforestry system			Rubber farmer
	Intensive agriculture	Oil palm production	Oil palm companies Independent smallholders		Plasma farmer Local community
	Forest harvesting	Timber production	Logging company	Government at district, provincial, and national level	Local community
		Non-timber forest product (NTFP)—Rattan collection		Government at district level	Rattan collector
		NTFP—Jelutong resin collection		Government at district level	Jelutong resin collector
Regulating	Climate regulation	CO_2_ sequestration		Government at district, provincial, and national level; Global community	Local community at the village

In this study, we selected six provisioning and one regulating services which are important for the livelihood of local people and the economic development in the district and the province. These seven ES include (1) timber production; (2) rattan collection; (3) jelutong resin (*Dyera costulata*) collection; (4) rubber (*Hevea brasiliensis*) production (based on permanent agroforestry system); (5) oil palm production on three management scales (company, plasma farmer and independent smallholder); (6) upland paddy production; and (7) carbon sequestration. In this study, we also include the analysis of nature recreation in Tanjung Puting National Park (Taman Nasional Tanjung Puting—TNTP) due to its importance for the livelihood of local people living around this national park. Further, we also include the analysis on orangutan habitat as a global concern.

### Valuation of Ecosystem Services

The benefits of the provisioning services in this study are assessed in monetary terms. We applied the valuation approach of ecosystem accounting (UN et al. 2014). Ecosystem accounting is the approach used to measure ecosystem capital in a way that is consistent with the national accounts (UN et al. 2014; Edens and Hein 2013). Ecosystem accounting involves an extension of the production boundary of the SNA to assess the capital of ecosystems based on their flow into economic and other human activities (UN et al. 2014; Hein et al. 2015). This approach allows for the inclusion of a broader set of ecosystem service types (i.e., regulating services) and the natural growth of biological assets in the accounts (UN et al. 2014).

In this research, we analyzed the net benefits of ES that are traded in the market (timber production, rattan collection, jelutong resin collection, agroforestry rubber production, oil palm production, and paddy production) expressed as an annual resource rent (RR). The annual RR has been valued by analyzing the market price and deducting the total costs (intermediate, employment, and user production cost) (Edens and Hein 2013). Considering the different time dimensions of the investment in ecosystem capital, we applied an ordinary annuity approach to calculate the annual RR of oil palm and agroforestry rubber production to make these services comparable. The annual RR was calculated from the net present value (NPV), which is the sum of the discounted revenues *R* minus cost *C*:

1 $${\text{NPV = }}\sum\limits_{t = 1}^{T} {\left( {R_{t} - C_{t} } \right)(1 + i)^{ - t} }$$

The NPV can be transformed into an annual payment *A:*

2 $$A = {\text{NPV}} \cdot \frac{{i(1 + i)^{T} }}{{(1 + i)^{T} - 1}}$$

where *A* is annual RR, *T* is the life time of the investment, and *i* is the discount rate, which is set at 10 % in our study (Based on Sumarga et al. (2015)).

In this study, we also analyzed the benefits of carbon sequestration (as the regulating service) based on the marginal social damage costs (Tol 2005) expressed as the social cost of carbon (SCC). The SCC is “an estimate of the monetized damages associated with the increment increase in carbon emissions in a given year” (Interagency Working Group on Social Cost of Carbon 2013). Since these marginal damage costs give a present value of future damage cost estimates, the discount rate plays an important role in determining the marginal damage costs. The SNA (UN et al. 2014) indicates that discounting should take place with market discount rates. In order to capture the public goods character of carbon damages, we apply a social discount rate of 3 % (Interagency Working Group on Social Cost of Carbon 2013). Consequently, we used an SCC value for 2010 at USD 32/ton CO_2_ that is equivalent to € 24/ton CO_2_ (€ 88/ton C) with an exchange rate of USD $ 1.33 for € 1 (average in 2010).

The main data and information used in this study were mostly obtained from the previous studies (2008–2010) and field work in 2012, as presented in Table 2. These secondary data include the information for economic analysis, the potential production of each service per year (yields), and macroeconomic parameters in 2010.

**Table 2:** Details of the data used in this study

Ecosystem service	Remark	Sources
Timber production	Financial report Performance of logging activities	Two logging companies; Setiawan et al. (2011)
Rattan collection	Economic analysis Potential yield/ha	Iwan (2008); Martoniady (2009)
Jelutong resin collection	Economic analysis Potential yield/ha	Sapiudin (2009); Budiningsih and Effendi (2013)
Agroforestry rubber production	Economic analysis Potential yield/ha	(Herman and Las 2009); Suyanto et al. (2009)
Upland paddy production	Economic analysis Potential yield/ha	Nugroho (2008); Yandi (2008)
Oil palm production	Economic analysis Potential yield/ha	Two oil palm companies; Iksan and Abdussamad (2010); Ismail (2010); Boer et al. (2012)
Carbon sequestration	Potential CO_2_ emission Social cost of carbon	Sanchez (2000); Agus et al. (2009); Hooijer et al. (2010); Lim et al. (2012); Carlson et al. (2012b); Carlson et al. (2012c); Interagency working group on social cost of carbon (2013); Agus et al. (2013); Gunarso et al. (2013)

### Allocation of Benefits to Different Types of Beneficiaries

**Fig. 2:**
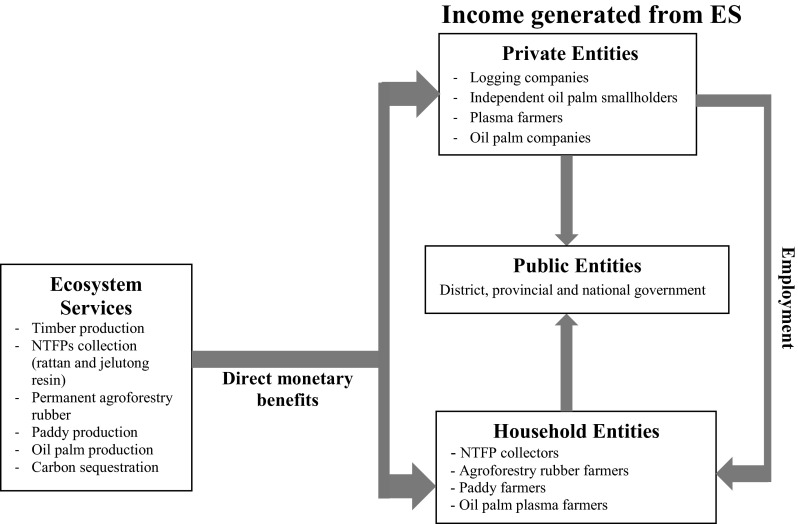
Income generated from ES by different groups of ES beneficiaries

Beneficiaries receive benefits from ES through different mechanisms. The allocation of benefits from ES received by beneficiaries was analyzed to explore the way benefits are shared between private, public, and household beneficiaries based on the framework presented in Fig. 2. The allocation of benefits to private entities was based on the annual net benefits. The allocation of benefits to household entities was based on annual benefits plus wages. The shares of the benefits public entities received from ES were calculated based on relevant public finance regulations applied at different levels of government. For instance, the share of benefits from timber production that public entities received at the district level was based on Government Regulation (PP) No. 55/2005 concerning the procedure for governing timber and non-timber forest products and Law No. 33/2004 concerning financial aspects of decentralization. These regulations determine taxes, including tax on timber and a land tax, and fees for extracting timber and non-timber forest products both from natural forests and plantation forests. Public finance regulations covered in this study are presented in Table 3.

**Table 3:** Legal framework in relation to taxes, provisioning, royalties, and benefit distribution

Service	Legally binding on public policies
Timber	Law (UU) No. 33/2004 Government Regulation (PP) No. 55/2005
Rattan	Government Regulation (PP) No. 55/2005
Jelutong resin	Government Regulation (PP) No. 55/2005
Permanent agroforestry rubber	Government Regulation No. 7/2007
Paddy	Government Regulation No. 7/2007
Oil Palm	Government Regulation (PP) No. 7/2007

### Potential Benefits in Different Land Uses

We explored the total monetary benefits private, public, and household beneficiaries received from different land uses. The total monetary benefits for each land-use type were derived from the sum of the monetary benefits beneficiaries received. The calculation of potential loss from carbon emissions was conducted based on marginal damage costs, capturing the cost of emitting a ton of carbon (CO_2_). We applied the social cost of carbon (SCC) value for 2010 at € 24/ton CO_2_, based on an assumed discount rate of 3 % (Interagency Working Group on Social Cost of Carbon 2013).

## Results

### Monetary Benefits Generated by Ecosystem Services

#### Provisioning Services

Compared to the other provisioning services that we analyzed, oil palm production provides the highest net benefit per ha; however, it also leads to significant societal costs related to CO_2_ emissions, in particular when oil palm is cultivated on peatland. Oil palm production on peatland generates an annualized resource rent for company, smallholder, and plasma farmer of € 683/ha/year, € 395/ha/year and € 451/ha/year, while on mineral soil, this is € 902/ha/year, € 537/ha/year, and € 765/ha/year, respectively. This difference reflects that the production costs, in particular for plantation establishment, are higher for peatland.

Timber production, both on peatland and mineral soil, generates a resource rent of, on average, € 30/ha/year. This relatively low value indicates that most of the forests in Central Kalimantan have been heavily logged in the past and that many of the commercial timber species (e.g., *Gonystylus bancanus* and *Eusideroxylon zwageri*) have already been harvested. The benefits from other forest products, in particular rattan and jelutong resin are considerably higher, on average, for the forest areas in Central Kalimantan, € 82/ha/year for rattan, and € 83/ha/year for jelutong. However, generation of the resource rent from these products is concentrated in the areas where there is active management and harvest of rattan or jelutong. In these areas, production can be as high as 1.3 ton/ha/year for rattan and 0.5 ton/ha/year for jelutong (in case enrichment planting of rattan or jelutong trees respectively has been carried out) (Sapiudin 2009). The resulting resource rent generated per ha, in these cases, amounts to € 110/ha/year for rattan, respectively, € 157/ha/year for jelutong.

Rubber production captured in this study is produced under a permanent agroforestry system with an average yield of about 0.67 ton/ha/year for the mineral soil and 0.54 ton/ha/year for the peatland (Suyanto et al. 2009). Agroforestry rubber production on the mineral soil provides a net benefit of € 112/ha/year, while production on peatland provides a net benefit € 47/ha/year.

Paddy is the most important food crop produced in this province. It is mainly grown by transmigrants who originally came from Java or Sumatra, although traditionally the Dayak ‘Ngaju’ in the provinces of Central and West Kalimantan have been practicing swidden rice cultivation for many generations (de Jong 1995). The average production of paddy in this area is about 2.2 ton/ha/year on the mineral soil and 1.7 ton/ha/year on the peatland. Paddy production generates an average resource rent of € 287/ha/year on mineral soil and € 184/ha/year on peatland. The large majority of paddy production in Central Kalimantan is used for local consumption.

Details of the results of the analysis on net benefits from provisioning services are presented in Table 4.

**Table 4:** Annual resource rent from provisioning services

Soil type	Ecosystem services	Yield (m3/ha/year; ton/ha/year)	Price (€/m3; €/ton)	Gross revenue (€/ha/year)	Production cost (€/ha/year)		Value added (€/ha/year)	Labor cost (€/ha/year)	Resource rent (€/ha/year)
					Intermediate cost (€/ha/year)	User cost of fixed assets (€/ha/year)			
Peatland	Timber	0.86	118	101	62	0	39	9	30
	Jelutong	0.28	342	96	6	0	90	7	83
	Rubber	0.54	500	270	7	0	263	216	47
	Oil palm (company)	19	123	1997	778	112	1107	424	683
	Oil palm (smallholder)	12	123	1278	403	164	711	316	395
	Oil palm (plasma farmer)	16	123	1697	701	189	807	356	451
	Paddy	1.7	238	405	80	6	319	135	184
Mineral soil	Timber	0.86	118	101	62	0	39	9	30
	Rattan	0.79	145	115	16	0	99	17	82
	Rubber	0.67	500	335	7	0	328	216	112
	Oil palm (company)	19	123	1997	637	84	1276	374	902
	Oil palm (smallholder)	12	123	1278	338	123	817	280	537
	Oil palm (plasma farmer)	16	123	1697	471	142	1084	318	766
	Paddy	2.22	238	528	87	7	434	147	287

#### Regulating Service (Carbon Sequestration)

The result of our analysis on monetary benefits from carbon sequestration shows that conversion of forest areas on the peatland and mineral soil to oil palm plantation provides the lowest benefits due to high CO_2_ emissions. Potential CO_2_ emission from converting forest to oil palm on the peatland is about 85 ton/ha/year and on mineral soil is about 25 ton/ha/year. The resulting monetary benefits generated per ha, in these cases, amount to € −2040/ha/year for the peatland and € −600/ha/year for mineral soil, respectively. These results show that converting forest area to oil palm plantation will increase the potential CO_2_ emission, which have become a global public concern. Detail on potential CO_2_ emissions and SCC analyzed in this study is presented in Table 5.

**Table 5:** Potential CO_2_ emissions and its Social Cost of Carbon (SCC)

Soil type	Land use	CO_2_ emission (ton/ha/year) (− indicates emission, + is sequestration)	SCC
Peatland	Forest	19.4	465.6
	Agroforestry	−14.4	−345.6
	Oil palm plantation	−85	−2040
	Agricultural land	−27.3	−655.2
Mineral soil	Forest	13.6	326.4
	Agroforestry	7.3	175.2
	Oil palm plantation	−25	−600
	Agricultural land	7.3	175.2

### Benefits from Employment

The monetary benefits beneficiaries receive from ES as employees or household entities are defined by the number of person working days per ha and wages per person per working day. In this study, we are concerned with farmers’ paddy and oil palm production (under plasma) based on the household system, which mainly ‘employs’ family members. Ecosystem accounting required deducting actual or implemented cost for labor, also in case the labor is provided by the farmer himself (UN et al. 2014; Obst et al. 2015). In order to keep the calculation in line with the ecosystem accounting methodology, we calculated the employment costs for these services based on the number of person days of family labor used per ha per year and multiplied this by the local average daily wage.

The results of our analysis show that in terms of the number of working days per ha, oil palm plantation under companies provides the highest number of person days (107 person days/ha/year), while timber production provided the lowest (0.7 person days/ha/year). On the other hand, in terms of wages, timber production provides the highest wages (€ 13/person/day), while the lowest wages are provided by paddy production (€ 3/person/day). The details of the benefits beneficiaries received for employment are presented in Table 6.

**Table 6:** Benefits beneficiaries received for employment

Ecosystem services	Number of person days/ha	Wages (€/person-day)	Wages (€/ha)
Timber	0.7	13	9
Rattan	3.1	5.4	17
Jelutong resin	1.2	5.8	7
Rubber	54	4	216
Paddy	49	3	147
Oil palm			
Smallholder	80	3.5	280
Plasma	91	3.5	318
Company	107	3.5	374

### Potential Net Benefits and Loss of ES Received by Beneficiaries from Different Types of Land Use

The change of forest to other land use will influence the supply of ES. Our analysis shows that the change of forest to other land use, particularly oil palm plantation, can potentially increase income for the sectors households and industry. However, it is important to note that within the household sector there may be important differences between costs and benefits accruing to different groups of people. For example, Dayak groups have in some cases sold (sometimes very cheaply) or lost their land to independent smallholders or oil palm companies. In this case, they have lost the opportunity of gaining benefits from other ES without adequate compensation, even though they may still receive benefits from oil palm production through employment However, not all local people can be employed on the plantations (McCarthy et al. 2012; Palupi 2014) and wages for casual labor are relatively low (€ 3.5/day; see Table 6).

The conversion of forest to oil palm plantation, particularly on the peatland, generates high CO_2_ emissions. Estimates for the CO_2_ emissions resulting from oil palm development on peatland range from 875 to 2125 ton/ha for the total period of 25 years, equal to 35–85 ton/ha/year (Herman and Las 2009; Agus et al. 2010; Hooijer et al. 2010; Lim et al. 2012; Carlson et al. 2012a, b; Couwenberg and Hooijer 2013). This results in social costs ranging from € −840/ha/year to € −2040/ha/year. On the other hand, a permanent agroforestry system on peatland may generate lower monetary benefits but also leads to much lower CO_2_ emissions. CO_2_ emissions from agroforestry systems strongly depend on type of agroforestry and drainage depth (if any drainage is applied). They vary from a small capture of carbon to net CO_2_ emissions of 14.4 ton CO_2_/ha (average from Agus et al. 2013; Sanchez 2000). We do not consider methane emissions from paddy fields in our study since all paddy fields in the study area are upland fields that do not cause methane emissions (Inubushi et al. 2003; Hadi et al. 2012). The results of our analysis on potential annual benefits received by private, public, and household entities, as well as potential losses due to the estimated CO_2_ emissions, are presented in Table 7.

**Table 7:** Potential monetary benefits from ES, its distribution to beneficiaries and environmental loss of land-use change

Soil type	Land use	Ecosystem services	Household (€/ha/year)				
			Production cost	Employment	Benefit sharing	RR	Total (RR + employment + benefit sharing)
Peatland	Forest	Timber		9	0.2^a^		9.2
		Jelutong	−6	7		83	90
		Carbon sequestration					
	Agroforestry	Rubber	−7	216		45	261
	Oil palm plantation	FFB (company)		424			424
		FFB (smallholder)		316			316
		FFB (plasma farmer)		356			356
	Agricultural land	Paddy production	−86	135		184	319
Mineral Soil	Forest	Timber		9	0.2^a^		9.2
		Rattan	−16	17		82	99
		Carbon sequestration					
	Agroforestry	Rubber	−7	216		112	328
	Oil palm plantation	FFB (company)		374			374
		FFB (smallholder)		280			280
		FFB (plasma farmer)		318			318
	Agricultural land	Paddy production	−84	289		147	436

Soil type	Land use	Ecosystem services	Private (€/ha/year)					Public (€/ha/year)				
			Production cost	Tax	Benefit sharing	Revenue	RR	Tax	Benefit sharing	CO_2_ emission (− indicates emission, + is sequestration) (ton CO_2_/ha/year)	SCC	Total
Peatland	Forest	Timber	−62	−17.8^a^	−0.2	101	30	17.8^a^		19.4	466	492
		Jelutong						9.6^a^				
		Carbon sequestration										
	Agroforestry	Rubber								−14.4	−346	−346
	Oil palm plantation	FFB (company)	−890	−26^b^		1997	683	26^b^		−85	−2040	−2014
		FFB (smallholder)	−567			1278	395					
		FFB (plasma farmer)	−890			1697	451					
	Agricultural land	Paddy production								−27.3	−655	−655
Mineral Soil	Forest	Timber	−62	−17.8^a^	−0.2	101	30	17.82		13.6	326	351
		Rattan						124				
		Carbon sequestration										
	Agroforestry	Rubber								7.3	175	175
	Oil palm plantation	FFB (company)	−721	−26^b^		1997	902	26^b^		−25	−600	−574
		FFB (smallholder)	−461			1278	537					
		FFB (plasma farmer)	−613			1697	766					
	Agricultural land	Paddy production								7.3	175	175

^a^Based on Government Regulation No. 55/2005

^b^Land acquisition, paid once for 25 years (Boer et al. 2012)

## Discussion

### Who Benefits from Ecosystem Services?

People obtain benefits from ecosystems in different ways. Our analysis of six provisioning and one regulating service in Central Kalimantan Province shows the monetary benefits received by different stakeholders. This study shows that upland paddy production provides the highest monetary benefits to household entities, while private and public entities receive most from oil palm and timber production. This study also shows how the monetary benefits from timber, NTFPs (rattan and jelutong resin), and agroforestry rubber are distributed to private, household, and public entities.

NTFPs and agroforestry rubber are the main source of local livelihoods in Central Kalimantan (Meijaard et al. 2013; Abram et al. 2014). However, the decrease in forest quality and agroforestry rubber areas has consequently decreased the stock of NTFPs and agroforestry rubber, and influences the monetary benefits received by household and public entities.

Oil palm production is a profitable venture in the case study area, in spite of fluctuations in market prices. Stakeholders have increasingly converted forest and agroforestry area to oil palm plantation, and have neglected the NFTPs and agroforestry rubber. The local government has seen oil palm plantation as an opportunity for economic development in their area through the increase in the number of jobs and also local people see it as an employment opportunity. In addition, the national target for CPO production has also supported this interest and caused an increase in the expansion of oil palm plantation in Indonesia.

The expansion of oil palm in Indonesia has been criticized locally and internationally. One of the criticisms in economic and social terms is related to the disadvantaged position of local communities when negotiating land transactions and business arrangements (Sirait 2009; McCarthy and Cramb 2009; Rist et al. 2010; Larsen et al. 2012; Obidzinski et al. 2012; Budidarsono et al. 2013; Dehen et al. 2013). An assessment of the characteristics of the private entities connected to oil palm production reveals that this activity is dominated by stakeholders with a high capital outlay, due to the high cost of establishing oil palm plantations. The cost of establishing an oil palm plantation in the first 3 years, on an independent smallholder scale, can be between € 428/ha/year and € 862/ha/year (Iksan and Abdussamad 2010; Boer et al. 2012). The break-even point can only be achieved with a minimum of 3 ha, assuming that smallholder farmers sell the fresh fruit bunches (FFB) at the farm gate (Boer et al. 2012; Budidarsono et al. 2013). Smallholders with the capital to establish oil palm are likely middle or upper class individuals with a close relationship with either an oil palm company or a key person at the district, provincial, or national level (Rist et al. 2010; Larsen et al. 2012; Dehen et al. 2013). Hence, the monetary benefits from oil palm production are mostly gained by companies and the elite with only a small share of the benefits going to the local communities and government through public regulations.

At the same time, some of the costs associated with palm oil production (traffic, road maintenance, and local externalities of oil palm plantations such as reduced access to the forest) occur at the district level. In addition, the rapid expansion of oil palm plantation in Central Kalimantan has also increased social conflicts associated with labor allocation (Rist et al. 2010; Dehen et al. 2013). Oil palm cultivation requires special skills that are more frequent among migrant smallholders with prior exposure to oil palm rather than for the local community with no prior experience. This has caused the exclusion of local people from this kind of work. The change in regulations governing partnerships in oil palm plantations, due to the establishment of Ministry of Agriculture Regulation No. 98/2013 that replaced Regulation No. 5/2011, has also created problems related to tenure and arrangements concerning plasma systems (McCarthy et al. 2012; Potter 2012). According to this new regulation, the plantations can no longer allocate 20 % of their concessions for plasma farming; they must find this outside their concession. This regulation is extremely difficult to implement in Central Kalimantan Province, since most of the recent transmigrants have become independent smallholders. Hence, plantations prefer to buy up Dayak land for inadequate levels of compensation to meet this regulation, which eliminates the opportunity for Dayak groups to receive other ES benefits, other than casual day labor (Palupi 2014). Problems related to environmental degradation have also increased due to the impact of oil palm expansion on deforestation, soil subsidence, hydrology, and climate change (Germer and Sauerborn 2007; Larsen et al. 2012; Yamamoto and Takeuchi 2012; Carlson et al. 2012b), see also the related work of Sumarga and Hein (2014) and Sumarga et al. (2015) in the same area.

### Potential Benefits and Losses When Changing a Forest Ecosystem to a Monoculture Plantation

ES trade-offs arise from management choices made by humans, who intentionally change the type, magnitude, and relative mix of services provided by an ecosystem. Trade-offs occur when the provision of one ecosystem service is reduced as a consequence of increased the use of another (Rodríguez et al. 2006). A common pattern of provisioning services is that they compete with each other (Tilman et al. 2002; Rodríguez et al. 2006). For example, an increase in oil palm production will reduce the timber and NTFPs production when oil palm is planted and replaces the forest.

Our analysis on potential benefits and losses in different land uses shows that the conversion of forest to oil palm plantation will increase the monetary benefits received by private and household entities, and decrease the monetary benefits received by public entities due to the absence of a regulation governing the FFB. The conversion of forest to oil palm plantation will also reduce the potential monetary benefits from nature recreation. Our interview with stakeholders in TNTP shows that this national park has generated the highest number of visitors (since visitors have been recorded) among all the national parks in Central Kalimantan. In 2010, the number of foreign visitors reached 8422 and domestic visitors 2343. The report from TNTP shows that in 2010, this national park has contributed € 612,578 to the local economy and € 51,471 to the national government (BTNTP 2012). However, the establishment of oil palm plantation around the buffer zone of this national park has become a salient issue that might reduce the environmental quality of TNTP and consequently influence the number of visitors. Our interviews with 50 boat operators and 150 tourists, during the period July to September 2012, also show the high concern about the water quality of the Sekoyer River. The reduction in water quality is due to the recent establishment an oil palm plantation (in 2011) in the buffer zone of TNTP. Most of the tourists (125 of 150) stated that they were upset about this environmental condition and most of the boat operators (35 of 50) thought that it would reduce the number of tourists visiting this national park in the future.

In environmental terms, converting forest to oil palm plantation will increase the environmental risk of deforestation, soil subsidence and carbon emissions, as well as decrease of biodiversity and the quality and quantity of river water (Germer and Sauerborn 2007; Hooijer et al. 2012; Agus et al. 2013; Azhar et al. 2014). As we show, the social costs related to CO_2_ emissions from oil palm on peat are higher than the total benefits private and public beneficiaries receive from oil palm production (cf. Sumarga et al. 2015).

The conversion of forest to oil palm plantation will also reduce the habitat of many endangered species such as the orangutan. The orangutan is an endangered species listed in appendix 1 of the convention on international trade in endangered species (CITES) for flora and fauna. It is Asia’s only remaining great ape, living only in Borneo and Sumatera (Nellemann et al. 2007). Moreover, Central Kalimantan is likely to have the world’s largest population of orangutan at the provincial level. The total population of wild orangutan in this province is about 33,000 individuals and 61 % of them occur in protected areas (Wich et al. 2008). Based on the unique place of Central Kalimantan as home to some 50 % of the remaining orangutan in the wild, maintaining the habitat for this species should be of special concern in particular in this province.

### Policy Implications

The establishment of policy instruments in natural resource management is vital when governing the distribution of ES benefits to private, public, and household entities. These instruments may not only ensure the sustainability of local livelihoods but also secure environmental funding that could be used to explore alternative and sustainable sources of financing ES management (Kettunen et al. 2009). For example, a reforestation fund from timber production could be used to cover reforestation costs of degraded forest areas.

Forest degradation and biodiversity loss has increased the awareness of the need to improve sustainable forest and land management in Indonesia. In response to that awareness, the government of Indonesia has released various regulations on sustainable forest management to govern the extraction of timber and NFTPs (including carbon sequestration), as well as nature recreation. The extraction of timber, both from natural forest and/or plantation forest, must be conducted according to certain regulations concerning reforestation funds, taxes on forest resources, and fees for concession permits. The national government also released a regulation governing the system for NTFPs collection and tariffs for entering a national park.

Considering the rapid deforestation and expansion of oil palm in Indonesia, it is very important to analyze the contribution of ES to forest ecosystems. Our analysis shows that timber and NTFPs have provided the highest benefits to public entities through Government Regulation No. 55/2005 on sustainable forest management. This regulation governs reforestation funds, taxes on forest resource, and fees for timber concession (both from natural forest and/or plantation forest) and NTFPs collection. However, the change in the value added tax (VAT) status of agricultural products in Government Regulation No. 12/2001 has eliminated any contribution from oil palm production to the public budget. In this regulation, FFB is listed as a non-taxable agricultural products, and the plantations (both companies and households) are only required to pay the cost of obtaining land cultivation rights (Hak Guna Usaha—HGU) of about € 208 to € 333/ha for 25 years and a land and building tax (PBB) of about € 10 to € 15/ha/year (Boer et al. 2012).

The public finance regulation applied to the palm oil sector is the tax on exporting CPO, kernel palm oil (KPO) and their derivative products. The export tax on these products is governed by the Ministry of Finance Regulation No. 67/Pmk.011/2010, based on Annex No II of the Ministry of Finance Regulation No. 223/Pmk.011/2008. The export tax is calculated in a progressive way, based on international prices of these products in cost, insurance and freight (CIF) Rotterdam. The export tax on CPO, KPO, and its derivative products is amended annually by the national government to increase the national revenue from the palm oil sector. However, this revenue is not distributed to the district and provincial governments. Considering the high cost of maintaining the infrastructure in the district, particularly roads (due to heavy loads transporting CPO and KPO), a request for a proportion of the income, from the import/export tax on CPO and KPO, to be directed to the producing district, was released by the Association of Indonesian District Government (Asosiasi Pemerintah Kabupaten Seluruh Indonesia—APKASI) at their meeting on 5 July 2014.

In order to support the sustainable production of agricultural products and address the environmental problems caused by the conversion of forest to monoculture plantations, there is a need to set up another policy instrument to govern the benefit distribution from the agricultural sector, particularly oil palm. This policy instrument should capture environmental aspects on sustainable oil palm production and secure the rights of local and poor people who depend heavily on forest ecosystems in which the forests area are converted to oil palm plantation. It is also important to revisit the financial regulation in this sector, to ensure that the monetary benefits received by public entities.

## Conclusions

This study assesses and analyzes the monetary benefits of seven ES in Central Kalimantan and how these benefits are allocated to different types of beneficiaries. This study shows that oil palm production provides the highest monetary benefits to private entities and lowest to public entities and local indigenous households, particularly Dayak groups. The benefits generated by this service are almost exclusively accrued by companies with at this point in time very little if any benefit sharing with local stakeholders, in particular when the local costs of oil palm expansion are considered. Considering oil palm plantation establishment as one driver of land-use change, there is a need to set up additional policy instruments to govern the sustainability of this product and to ensure that the monetary benefits are received by public entities through a tax schedule. This policy instrument should reflect the environmental indicators for sustainable palm oil production and secure the rights of local and poor people who depend heavily on forest ecosystems. In addition, it is also important to link up with the international carbon system in securing the economic incentives under REDD^++^ schemes, particularly if the government and communities decide to conserve forest instead of converting them to oil palm plantation.
